# Reconstructing and forecasting the COVID-19 epidemic in the United States using a 5-parameter logistic growth model

**DOI:** 10.1186/s41256-020-00152-5

**Published:** 2020-05-15

**Authors:** Ding-Geng Chen, Xinguang Chen, Jenny K. Chen

**Affiliations:** 1 School of Social Work, University of North Carolina, Tate-Turner Kuralt Building 548-C, CB #3550, Chapel Hill, NC 27599 USA; 2 Department of Statistics, University of Pretoria, Pretoria, South Africa; 3 Department of Epidemiology, University of Florida, Gainesville, USA; 4 Department of Statistics and Data Science, Cornell University, Ithaca, USA

**Keywords:** COVID-19, Epidemics, Disease dynamics, Population-based model, Logistic growth model, Prediction, Reconstruction, Under-detection, Tipping point, USA

## Abstract

**Background:**

Many studies have modeled and predicted the spread of COVID-19 (coronavirus disease 2019) in the U.S. using data that begins with the first reported cases. However, the shortage of testing services to detect infected persons makes this approach subject to error due to its underdetection of early cases in the U.S. Our new approach overcomes this limitation and provides data supporting the public policy decisions intended to combat the spread of COVID-19 epidemic.

**Methods:**

We used Centers for Disease Control and Prevention data documenting the daily new and cumulative cases of confirmed COVID-19 in the U.S. from January 22 to April 6, 2020, and reconstructed the epidemic using a 5-parameter logistic growth model. We fitted our model to data from a 2-week window (i.e., from March 21 to April 4, approximately one incubation period) during which large-scale testing was being conducted. With parameters obtained from this modeling, we reconstructed and predicted the growth of the epidemic and evaluated the extent and potential effects of underdetection.

**Results:**

The data fit the model satisfactorily. The estimated daily growth rate was 16.8% overall with 95% CI: [15.95, 17.76%], suggesting a doubling period of 4 days. Based on the modeling result, the tipping point at which new cases will begin to decline will be on April 7th, 2020, with a peak of 32,860 new cases on that day. By the end of the epidemic, at least 792,548 (95% CI: [789,162, 795,934]) will be infected in the U.S. Based on our model, a total of 12,029 cases were not detected between January 22 (when the first case was detected in the U.S.) and April 4.

**Conclusions:**

Our findings demonstrate the utility of a 5-parameter logistic growth model with reliable data that comes from a specified period during which governmental interventions were appropriately implemented. Beyond informing public health decision-making, our model adds a tool for more faithfully capturing the spread of the COVID-19 epidemic.

## Introduction

Coronavirus disease 2019 (COVID-19) is an infection caused by a novel pathogen named SARS-Cov-2. Spreading worldwide in less than five months, the COVID-19 pandemic is a typical example of a global health issue [1]. In the months since the first COVID-19 case was reported in the United States on January 22, 2020, many studies have employed different models to reconstruct the epidemic (i.e., the spread of COVID-19 within the United States only) and forecast its future trends, from simple growth models to classic susceptible-infected-recovered models [2]. Yet due to the scarcity of available information about the early period of the COVID-19 epidemic, researchers lack sufficient data to construct complex and classic epidemiological models. In this context, the population-based ecological growth model is the preferable option for predicting the epidemic’s future trajectory.

Researchers have developed various population-based models for modeling population dynamics and disease epidemics. One such model is the 1-parameter exponential growth model. In this model, population growth has no upper limit and is determined by one parameter of growth rate. To account for the upper limit of population growth, the 2-parameter logistic growth model was developed. In this model, the population growth rate is exponential in the beginning, but this growth rate gets smaller and smaller as population size approaches a maximum carrying capacity as detailed described in Richards [3], McIntosh [4], Renshaw [5], Kingsland [6], and Vandermeer [7].

To account for additional key characteristics of population growth, the 2-parameter logistic growth model has since been extended to 3-parameter, 4-parameter, and 5-parameter logistic growth models. These models have been widely used in other fields of research, including demography and analytical chemistry [8, 9]. Despite the many analytical advantages of these models, to our knowledge, no study has employed this 5-parameter logistic growth model to examine the COVID-19 epidemic in the United States or in other countries. Thus, one purpose of this study is to assess the utility of the 5-parameter growth model in studying the dynamics of the spread of COVID-19.

Unlike typical population growth models (in which the initial population is a known quantity), only a small number of COVID-19 cases were detected during the early phase of the epidemic in the United States. In all contexts, more extensive testing services detect more cases; when the initial time of an epidemic’s outbreak is known, extensive testing can yield data that more accurately reflects the true growth of the epidemic. Data indicate that the incubation period of COVID-19 is about 14 days [10], and COVID-19 testing services in the U.S. became available in mid-March and were sustained thereafter following CDC guidelines. Therefore, the 14-day interval following the widespread implementation of testing should demonstrate the highest level of detection rates unaffected by the removal of infected individuals from the growth curve, presenting ideal data for model building. In principle, a model built with this data would more accurately capture and predict the growth of COVID-19 than models constructed from infection data ranging from the first detected case to the present.

## Methods

### Data

Data for this study were the daily cumulative cases of COVID-19 in the U.S. from January 22 to April 6, 2020. This real-time data were compiled by the Centers for Disease Control and Prevention (CDC) and made available on their website at the time we conducted our study [11].

### Models

We modeled the data using the 5-parameter logistic growth model as below:

1 $$ C(t)={C}_{min}+\frac{C_{max}-{C}_{min}}{{\left[1+{e}^{-r\left(t-{t}_{mid}\right)}\right]}^{\alpha }\ } $$

where

1. *C*(*t*) is the number of cumulative cases of COVID-19 over time, *t* (*t* = 1/22/2020, 1/23/2020, …, 4/6/2020);
2. *C*_*min*_ is the minimum number of cases at the beginning of the epidemic on January 22, 2020, when the first case was reported in the U.S.;
3. *C*_*max*_ is the maximum number of people infected by the time the epidemic ends (i.e. the model-predicted total number of Americans who will be infected with COVID-19);
4. *r* is the daily exponential growth rate;
5. *t*_*mid*_ is the estimated tipping point when the number of new daily cases begins to level off and then to decrease; and
6. *α* is an asymmetric parameter quantifying the skewness of the distribution of daily new cases. *α* = 1 indicates a symmetric distribution centered at *t*_*mid*_; *α* > 1 indicates faster increases in new cases before *t*_*mid*_ and slower after *t*_*mid*_; and the reverse if *α* < 1.

With Model 1 defined above, daily new cases *D*(*t*) can be obtained by taking the first derivative of the model:

2 $$ D(t)={C}^{\prime }(t)=\frac{\alpha r\left({C}_{max}-{C}_{min}\right)}{{\left[1+{e}^{-r\left(t-{t}_{mid}\right)}\right]}^{\alpha +1}} \times {e}^{-r\left(t-{t}_{mid}\right)}+\in (t), $$

where the error term ∈(*t*) is assumed to be normally distributed with mean 0 and standard deviation of σ.

### Implementation of modeling analysis

We conducted our data analysis using the software R. A 5-parameter logistic growth model was fitted to the data for new daily infections from March 21, 2020 to April 4, 2020, as shown in Model 2. Using the R function “optim,” we implemented modeling analysis using a nonlinear optimization algorithm to minimize the sum of squared errors between the observed and model-estimated data. The optimization process yielded estimates for the five parameters *C*_*min*_, *C*_*max*_, *t*_*mid*_, *r*, and *α* with a significance level set at *p* <  0.05 (two-sided).

With these five estimated model parameters, we estimated model-based cumulative cases (using Model 1) and new cases (using Model 2) for each day from March 21 to April 4 and made predictions about cumulative and new daily cases after April 4. We calculated the underdetection of cases in this 2-week window by measuring the differences between the reported number and the model-predicted number of cases.

## Results

Model 2 fitted the observed cumulative daily cases from March 21 to April 4 satisfactorily and the model fit converged nicely. Table 1 summarizes the estimated parameters, their standard error (SE), and their 95% confidence intervals (CI). Except for *C*_*min*_, all model parameters were statistically significant at *p* <  0.001 level. The lack of significance for *C*_*min*_ appears to be reasonable given the small scale of this number relative to the other parameters and the practical difficulties of determining the number of actual cases at the beginning of the epidemic when the first few COVID-19 cases were detected and reported.

**Table 1:** Summary of parameter estimation

Parameter	Estimate	SE	*p*-value	Lower 95% CI	Upper 95% CI
***C***_***min***_	29.999	2059.86	0.988	− 4007.33	4067.32
***C***_***max***_	792,548	1727.56	< 0.0001	789,162	795,934
***t***_***mid***_	76.9	0.456	< 0.0001	75.952	77.739
***r***	0.16854	0.00463	< 0.0001	0.15947	0.17761
**α**	0.95364	0.06194	< 0.0001	0.83224	1.07504

Note: Parameters were estimated based on daily cases of COVID-19 in the U.S. between March 21, 2020 and April 4, 2020

Based on our model estimates, at least 792,548 (95% CI: [789,162, 795,934]) Americans will have been infected with COVID-19 by the time the epidemic ends. This number is slightly more than twice the number of infections that had occurred in the U.S. by April 6. For reasons we discuss later, this estimate may be conservative, as the total number of reported cases exceeded 800,000 on April 21, as we completed our revisions of this paper.

Our estimated tipping point for new daily cases was on about April 7, 77 days (95% CI: [76, 78]) from the beginning of the epidemic on January 22. In other words, our model predicted that the epidemic curve in the U.S. would begin to flatten around April 6–8, 2020. This estimation corroborates recent reporting that new daily cases in the U.S. have remained somewhat constant beginning in early April [12]. This tipping point suggests that it will take three to four more COVID-19 incubation periods (i.e., 6 to 8 weeks) for the U.S. to bring the epidemic under control, given our documentation and analysis of this process in China [10] (Chen X, Yu B, Chen D: Three month of COVID-19 in China: surveillance, evaluation, and forecast from outbreak to control with a second derivation model, submitted).

The estimated exponential daily growth rate of COVID-19 in the U.S. population is 16.9% (95% CI: [15.9, 17.8%]), nearly the rate observed in China (17.12%) [10]. This U.S. rate suggests that the number of total COVID-19 cases in the U.S. will double every four days if no anti-epidemic actions are in place. The estimated asymmetric parameter α was 0.954 (95% CI: [0.832, 1.075]), which is not statistically different than α = 1.0. This result indicates that changes in COVID-19 cases before and after the predicted tipping point of April 7 will follow a similar pattern.

For further illustration, Table 2 summarizes three sets of information ordered by days from the beginning of the epidemic: the data used for the model fitting section, a smaller reconstruction section, and a prediction section. Our fitted model detected substantial underdetected COVID-19 cases. By April 7, when this study was completed, the CDC reported a total of 395,011 detected cases; with our model, we predicted that CDC data for reported cases in fact underreported about 19,291 cases up to April 9.

**Table 2:** Illustration of data usage with reported, predicted, and underreported counts

Data Usage	Days	Date	Reported Cases		Predicted		Under-reported
			Total	Daily	Daily	Total	
**Reconstruction**	54	3/15/2020	3487	1253	3108	19,781	16,294
	55	3/16/2020	4226	739	3623	23,141	18,915
	56	3/17/2020	7038	2812	4218	27,054	20,016
	57	3/18/2020	10,442	3404	4902	31,606	21,164
	58	3/19/2020	15,219	4777	5687	36,892	21,673
	59	3/20/2020	18,747	3528	6584	43,019	24,272
**Fitting**	**60**	**3/21/2020**	**24,583**	**5836**	**7603**	**50,102**	**25,519**
	**61**	**3/22/2020**	**33,404**	**8821**	**8755**	**58,269**	**24,865**
	**62**	**3/23/2020**	**44,183**	**10,779**	**10,047**	**67,658**	**23,475**
	**63**	**3/24/2020**	**54,453**	**10,270**	**11,485**	**78,411**	**23,958**
	**64**	**3/25/2020**	**68,440**	**13,987**	**13,070**	**90,676**	**22,236**
	**65**	**3/26/2020**	**85,356**	**16,916**	**14,797**	**104,598**	**19,242**
	**66**	**3/27/2020**	**103,321**	**17,965**	**16,656**	**120,315**	**16,994**
	**67**	**3/28/2020**	**122,653**	**19,332**	**18,624**	**137,947**	**15,294**
	**68**	**3/29/2020**	**140,904**	**18,251**	**20,670**	**157,589**	**16,685**
	**69**	**3/30/2020**	**163,539**	**22,635**	**22,750**	**179,298**	**15,759**
	**70**	**3/31/2020**	**186,101**	**22,562**	**24,810**	**203,082**	**16,981**
	**71**	**4/1/2020**	**213,144**	**27,043**	**26,784**	**228,889**	**15,745**
	**72**	**4/2/2020**	**239,279**	**26,135**	**28,600**	**256,597**	**17,318**
	**73**	**4/3/2020**	**277,205**	**37,926**	**30,180**	**286,010**	**8805**
	**74**	**4/4/2020**	**304,826**	**27,621**	**31,453**	**316,855**	**12,029**
**Forecast**	75	4/5/2020	330,891	26,065	32,352	348,791	17,900
	76	4/6/2020	374,329	43,438	32,830	381,419	7090
	77	4/7/2020	395,011	20,682	32,860	414,302	19,291
	78	4/8/2020	427,460	32,449	32,436	446,987	19,527
	79	4/9/2020	459,165	31,705	31,582	479,030	19,865
	80	4/10/2020	492,416	33,251	30,340	510,021	17,605

Using a 2-week interval (i.e., March 21 to April 4) of data, our model’s prediction of the number of new daily cases from April 5 to April 11 matched quite well with the observed data. For example, the model-predicted number on April 9 was 31,705, very close to the observed number of 31,582.

These results should be interpreted with caution. The estimated sum square of error $\hat{\sigma}$ = 2638.434 is quite large, meaning that although our model fitted the 2-week interval of data very well, a large amount of variation in the data is not explained by this model.

**Fig. 1:**
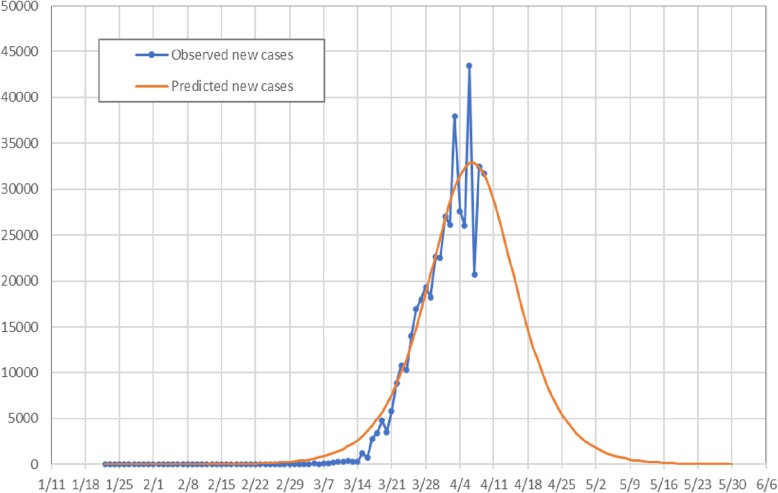
Observed vs. model-estimated and forecasted daily new COVID-19 cases, January 22–May 30, U.S.A

**Fig. 2:**
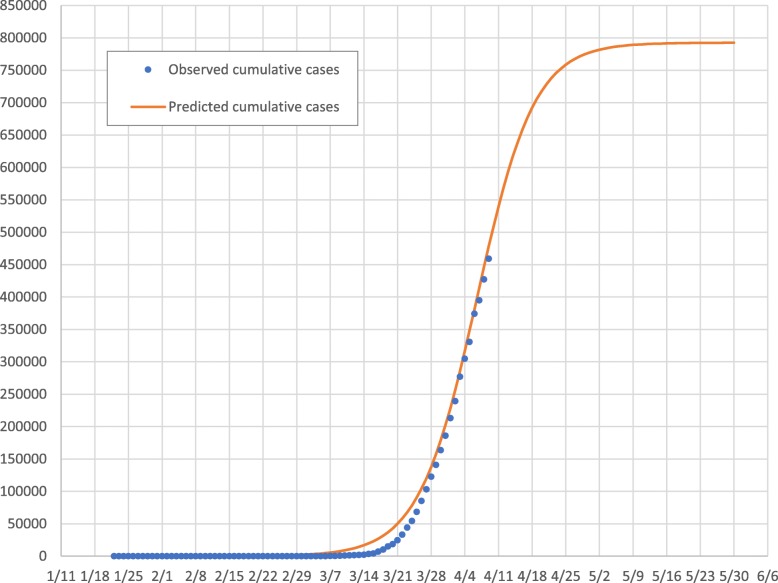
Observed vs. model-estimated and forecasted daily cumulative COVID-19 cases, January 22–May 30, U.S.A

Below we provide two figures comparing the observed and model-predicted dynamics of new daily cases (Fig. 1) and of cumulative cases (Fig. 2). Overall, the model we constructed from only two weeks of data very closely predicted the reported numbers of both new and cumulative cases. Correspondingly, our model predicts that the cumulative cases will continue to increase rapidly after the tipping point until early May, as illustrated in Fig. 2.

## Discussion

This study details our efforts to model, reconstruct, and forecast the COVID-19 epidemic using a 5-parameter logistic growth model – a method widely used in demography, biology, and other hard sciences. To our knowledge, we are the first to use this model to analyze the COVID-19 epidemic in the U.S. We also developed and used our model through an innovative approach. Namely, to fit the model we intentionally used data from a 2-week period when new cases could be more completely detected, and we then used this fitted model to reconstruct the growth of cases before and after the 2-week period as well as to forecast the future development of the epidemic beyond the study period.

Based on findings from our modeling analysis, there is not a high likelihood that the number of daily new cases will increase continuously after the tipping point (i.e., April 7, 2020). However, our model’s estimation that at least 800,000 Americans will be infected over the course of the epidemic may be conservative, given that the total number of reported cases exceeded 800,000 on April 21, as we completed our revisions of this paper, while the new cases fluctuated between 26,000 and 35,000 per day due to the increased appearance of cases in other cities and states outside of New York.

This conservative estimation is potentially attributable to three factors. First, the exponential growth of our logistic model is very sensitive to differences in growth rate, and a small difference in the number of early cases can lead to a sizeable difference in predictions of subsequent cases. Second, although we strategically selected a 2-week interval of data that we believed would yield the best model for predicting the epidemic’s growth, this data likely still underreported the actual number of COVID-19 infections, making our estimated growth rate smaller than the true growth rate. For example, the estimated exponential growth rate of COVID-19 is 17.12% for China [10], higher than 16.85%, the rate we estimated for the U.S. A small difference in the exponential growth rate can result in substantial differences in the maximum number of infections. And third, the data used for this analysis is from March 21 to April 4, 2020, where most of the reported cases are from the states of New York and New Jersey. The reported cases from these two states are flattened from reported CDC. Still, more cases are reported from other states, especially from the states of Michigan, Florida, Louisiana, which would add to the cases from New York and New Jersey to exceed the 800,000 predicted.

The accuracy of our model is also contingent on the federal- and state-level policy decisions that emerge in coming months. Although many states have implemented strict shelter-in-place policies to slow down the epidemic’s spread, several states still have no such policies in place. In the absence of further policy action, we expect that more cases will be reported which may greatly surpass the estimated 800,000, and that the actual infection tipping point may occur later in April. Indeed, significant variations still persist in the estimated total infections in the U.S. even in light of available data: Ferguson et al. [13] predicted 2.2 million cases whereas the CDC’s worst-case scenario model predicted a shocking 214 million cases [14]. At this moment, it remains unclear which estimates are more reliable. The accuracy of our estimation will be tested in light of emerging data on the progression of the epidemic in the United States.

The daily exponential growth rate of COVID-19 is 16.85% for the U.S. population, nearly the rate observed in China (17.12%) [10]. Daily exponential growth rates can be obtained with limited data in the early period of an epidemic, and they provide a dynamic measure of instantaneous change, making doubling times calculated based on growth rate highly useful for directing and evaluating anti-epidemic measures. The U.S.’s daily exponential growth rate suggests that the number of COVID-19 infections will double every four days. For example, if the total cases are 500,000 today, there will be 1,000,000 in four days (with 40,000 anticipated deaths) if no timely anti-epidemic measures are implemented. No one – including policymakers, medical and health professionals, and the general public – should ignore this evidence of the pressing need to control the pandemic.

## Conclusion

Understanding and curbing the COVID-19 epidemic in the U.S. is an essential part of fighting the pandemic globally [1]. This study provides data important for informing public health decision-making designed to end the epidemic in the U.S. Our study also demonstrates the utility and efficiency of the 5-parameter logistic growth model for examining the dynamics of an epidemic in its early period when little data is available. Additionally, our selection of the 5-parameter logistic exponential growth model was based on intensive testing of other models, including 2-parameter, 3-parameter, and 4-parameter models. Of all models tested, the 5-parameter produced the most accurate results and generated key information, including the exponential growth rate, the doubling time for the epidemic, and the tipping point when daily new cases will level off.

Our study’s findings should be considered in light of their limitations. First, our strategic selection of data from a specific timeframe is more subjective than objective, and not applicable in all contexts. Researchers applying this method in different countries/regions with different anti-epidemic strategies implemented in different ways should make their own determinations regarding the optimal timeframe to select for their modeling. We selected the 2-week interval from March 21 to April 4 because this interval spans approximately one COVID-19 incubation period and because the U.S. government began implementing widespread testing services by the beginning of this period, meaning that data from this interval potentially captured a more representative set of new cases. Interested readers can conduct their own analyses using this model while expanding on this time window to further assess the utility of this method. So far, the model’s short-term predicted daily cases are quite close to the observed daily cases, as shown by Table 2. However, our model’s long-term predictions of future new daily cases may not be accurate (which is true of any model-based long-term prediction), so these long-term predictions should be considered with caution.

Second, additional work is needed to improve confidence in the accuracy *C*_*min*_, the minimum number of cases at the beginning of an epidemic. It is challenging to improve this estimation given the large range of different measures in the model. For example, the range between *C*_*min*_ and *C*_*max*_ in our analysis is from about 30 to about 800,000. Furthermore, the number of reported cases at the beginning of the epidemic is highly unreliable due to a lack of testing protocols and perhaps a lack of awareness of the incipient epidemic itself, which will lead in turn to an unreliable estimation of *C*_*min*_.

Despite the limitations, findings from this study provide timely data that can inform public health decision-making and policies designed to end the epidemic. We will continue to update our model as more data become available and the COVID-19 epidemic in the United States continues to evolve.

## Data Availability

The dataset supporting the conclusions of this article is available from the Centers for Disease Control website, https://www.cdc.gov/coronavirus/2019-ncov/cases-updates/cases-in-us.html.

## Abbreviations

CDC: Centers for Disease Control and Prevention
CI: Confidence interval
COVID-19: Coronavirus disease 2019
SE: Standard error
